# Molecular testing for acute myeloid leukemia

**DOI:** 10.20892/j.issn.2095-3941.2020.0734

**Published:** 2022-01-15

**Authors:** Dahui Qin

**Affiliations:** 1 Moffitt Cancer Center, Tampa, FL 33612-9416, USA

**Keywords:** Molecular, test, next-generation sequencing, acute myeloid leukemia

## Abstract

In the era of personalized medicine, information on molecular change at the gene level is important for patient care. Such information has been used for disease classification, diagnosis, prognosis, risk stratification, and treatment, which is especially important in cancer patient care. Many molecular tests exist and can be used to detect the molecular changes at gene level. These tests include, but are not limited to, karyotyping, endpoint polymerase chain reaction (PCR), real-time PCR, Sanger sequencing, pyrosequencing, next-generation sequencing, and so forth. How to use the right tests for the right patients at the right time is essential for optimal patient outcome. This review puts together some information on molecular testing for acute myeloid leukemia.

## Introduction

Acute myeloid leukemia (AML) is a clonal malignant neoplasm of myeloid cell lineage involving the blood and bone marrow, but other tissue can also be occasionally affected^1^. In the era of personalized precision medicine, molecular changes have been used in AML classification, diagnosis, prognosis, risk stratification, and treatment^1,2^. In the 2016 World Health Organization AML classification, molecular changes have been extensively incorporated into the classification. AML has been classified as AML with recurrent genetic abnormalities, AML with myelodysplasia-related changes, therapy related myeloid neoplasm, AML not otherwise specified, and myeloid sarcoma^1^. It is now understood that recurrent genetic abnormalities in AML can be classified into two categories: one category has chromosomal rearrangements, resulting in translocations and fusion genes, and the other category of recurrent genetic abnormalities is characterized by gene mutations such as point mutations, deletions, and insertions. Copy number variations is also getting attention^3^.

### AML with chromosomal rearrangements (translocations)

Different recurrent translocations have been identified in AML. The translocations include, but are not limited to t(15;17)(q24.1;q21.2), t(8;21)(q22;q22.1), inv(16)(p13.1 q22) or t(16;16)(p13.1;q22), t(9;11)(p21.3;q23.3), t(6;9)(p23;q34.1), inv(3)(q21.3 q26.2) or t(3;3)(q21.3;q26.2), and t(1;22)(p13.3;q13.1)^1,4^. Identifying these translocations in the process of AML diagnosis is critically important. For example, identifying t(15;17) translocation is important for the diagnosis of acute promyelocytic leukemia (APL). Such translocation results in the *PML–RARA* fusion gene, which has implications in the prognosis and offers specific treatment options.

### AML with gene mutations

Different recurrent gene mutations have been identified in AML. Such changes include, but are not limited to, mutations in in the *NPM1*, *CEBPA*, *RUNX1*, *DNMT3a*, *FLT3*, *KRAS*, *NRAS*, *ASXL1*, *IDH1/IDH2*, *TET2*, *U2AF1*, *SRSF2*, *KMT2A*, *TP53*, and *WT1* genes. Identifying any of such mutations can also be important in AML diagnosis, prognosis, and treatment. For example, identifying *FLT3* mutations implicates possible unfavorable disease progress. Meanwhile, it may offer specific treatment using *FLT3* inhibitors. Molecular testing in AML is a fast-evolving area. New gene mutations have been identified every year^5^. Molecular testing has been widely used in characterizing different entities in AML with recurrent genetic abnormalities and is playing an important role in AML patient care. Different molecular testing methods are being used.

## Methods of molecular testing

Molecular testing has been defined in different ways. The broad definition may include any testing that reveals DNA changes. Under such broad definition, karyotyping, fluorescence *in situ* hybridization (FISH), polymerase chain reaction (PCR)–associated testing, traditional sequencing, and next-generation sequencing (NGS) are all considered as molecular testing. In a narrower definition, molecular testing is defined as any testing that reveals the changes in the nucleotide in a DNA and RNA sequence. Such tests include PCR-associated molecular testing, traditional sequencing, and NGS. As more and more different technologies evolve, the line between the broad definition and narrow definition starts to blur. However, regardless of the definition, different molecular tests are important in identifying molecular changes in AML for diagnosis, prognosis, risk stratification, and treatment decision. This review will focus on the requirements of AML testing and the methods needed to address those requirements.

Generally speaking, clinical assessment of AML starts from clinical manifestations, such as fatigue, bleeding, immunodeficiency, anemia, leukopenia, leukocytosis, thrombocytopenia, thrombocytosis, and so forth. The pathology evaluation of AML starts from the morphological analysis of the changes in bone marrow and peripheral blood, but other anatomic locations may also be investigated. The focus is usually bone marrow myeloblast count. Twenty percent of the blast in bone marrow or in blood is usually used as one of the criteria for AML diagnosis, but molecular genetic changes should be taken into consideration, which may lower the blast criteria to less than 20%^1^. As for AML molecular testing, the 2017 European LeukemiaNet (ELN) and the NCCN 2019 guidelines recommend karyotyping and FISH test for AML patients^6,7^. Different testing methods are used for AML subclassification, risk stratification, and treatment decision.

### Karyotyping

Karyotyping is a traditional cytogenetic technology. It has been widely used for cytogenetic assessment of different diseases, including AML. Such assay needs to culture the cells from a sample and usually in the presence of a certain mitogen. The proliferating cells will be stopped at the metaphase. The morphology of the metaphase chromosomes will be analyzed to obtain karyotyping result. In the 2017 ELN recommendations, conventional cytogenetic analysis (karyotyping) is still recommended for all AML cases. Karyotyping can identify many recurrent translocations in AML leukemic cells, which are used in AML classification. Karyotyping can also identify complex chromosomal changes. These changes are used in risk stratification of AML. Currently, karyotyping is a very useful technique. However, karyotyping has its limitations. First of all, the cells in a given sample have to proliferate to result in metaphase chromosomes for analysis. If the targeted cells fail to proliferate, the metaphase chromosomes of those cells will not be available for analysis. Therefore, the karyotyping result may be biased. Additionally, sometimes, translocation involves a small region of the chromosome that is beyond the resolution of the microscopic karyotyping and, therefore, cannot be identified by karyotyping. Such changes are called cryptic chromosomal changes, and they need to be detected by a different method, for example, FISH.

### FISH

FISH has been used in identifying translocations. FISH assay uses probes, which consist of a segment of DNA conjugated with a fluorophore. The probe DNA sequence is complementary to a segment of genomic DNA to be interrogated. The probe DNA is usually hundreds of kilobase long. When it binds to a segment of targeted genomic DNA, the fluorophore conjugated with it can be visualized under fluorescence microscope. The FISH probes can be centromere-specific probes, which can be used to identify chromosomes. The FISH probes can be locus-specific, which can be used to identify specific gene locus. Therefore, FISH assay can be used to identify chromosome translocations and most cryptic chromosome translocations. FISH can be done on metaphase and interphase targets. It is usually more sensitive than karyotyping.

### PCR-associated molecular testing

PCR-associated molecular testing methods take advantage of the PCR amplification to make more copies of targeted DNA or RNA. Therefore, PCR-associated assays are usually more sensitive. The specificity of these assays is determined by the primers and probes used in such assays. The primers and probes in these assays are usually short segments of oligonucleotides, usually 10 to 30 bp long, which is much shorter than the FISH probes. Therefore, compared with FISH assay, PCR-associated assays can interrogate more specific genomic DNA changes with higher resolution. However, such assays have their own drawbacks. For example, some translocations may have numerous break points. One will have to design many pairs of primers for each of these break points, which can make PCR assays less practical for certain types of translocations.

### Traditional sequencing

Sanger sequencing is a widely used traditional sequencing method. It is also used in AML case assessment. It can identify point mutations and insertions/deletions. However, its detection limit in terms of allelic frequency is approximately 15%. When a mutant gene in a sample is less than 15%, Sanger sequencing may not be able to detect it, resulting in false-negative results. Pyrosequencing is another sequencing method, which can have a sensitivity of approximately 5%, depending on the types of mutations. Therefore, it has some advantage over Sanger sequencing in terms of sensitivity. It is also relatively easier for pyrosequencing to quantitate variant allelic frequency. However, pyrosequencing also has its own drawbacks. Usually, it can only interrogate relatively shorter length of DNA sequence. It is usually designed to interrogate a segment of DNA sequence less than 20 bp long, whereas Sanger sequencing can interrogate a sequence of hundreds of base pair long.

### NGS

NGS is a revolutionized sequencing technology. Combining massive parallel sequencing chemistry and bioinformatics, it is able to interrogate hundreds and thousands of genes or even whole genome in one test. NGS has changed the landscape of molecular testing in disease diagnosis, treatment, and patient follow-up management. It is also changing the landscape of disease classifications.

There are different designs of NGS assays. In terms of scale, it can range from whole-genome sequencing to whole-exome sequencing or disease-specific gene group sequencing (NGS panel assay), focusing on a group of genes associated with certain types of neoplasms, like myeloid NGS panel or solid tumor NGS panel. The advantage of whole-genome sequencing is that it has the widest coverage of the genome. It can help discover previously unknown genomic changes, and it is more widely used in research. It can also be used in clinical settings. For example, it has been used in constitutional genetic disease diagnosis, even in intensive care units^8^. The cost of whole-genome sequencing is relatively high. The sequencing depth is limited compared with small NGS panels. Some variants with low variant allele frequency (VAF) may not be detected due to limited sequencing depth. Whole-exome NGS assay mainly focuses on the coding region of the genome. As the targeted area narrows, the assay allows more sequencing depth. It has been used more in clinical applications. However, the most common clinical NGS assays are specific NGS panel assays, which usually focuses on dozens or hundreds of disease-related genes. Compared with whole-exome NGS assay, NGS panels further narrow the targeted areas. Therefore, NGS panel assay allows more sequencing depth, easily reaching hundreds or thousands of average reads. With such sequencing depth, an NGS panel assay is able to detect mutants with relatively low VAF. The sensitivity usually reaches 5%. From the test sample aspect, NGS assay can be designed to test DNA or RNA or a combination of both. DNA NGS assays are more often used to interrogate point mutations, short insertions, and deletions. RNA NGS assays are more useful in detecting fusion gene changes; however, DNA and RNA assay function can overlap and complement each other. From methodology aspect, there are amplicon NGS assays, hybridization capture NGS assays, or combined assays taking advantage of both amplicon and hybridization capture assays. In recent years, NGS assay has been used in monitoring minimal (measurable) residual disease (MRD). Using unique molecular identification (UMI) barcode, NGS sequencing error and noise background have been reduced, which makes NGS assay a good tool in monitoring MRD^9–16^. NGS assay has also been used in single cell sequencing, which is a powerful tool for revealing information about clonal evolution^17,18^. NGS has been widely used in clinical service and provided genomic information that we have never seen before.

### Other technologies

There are other technologies that have been used in molecular testing. Spatial transcriptomic technology combines molecular tests with microscopic location, which enables us to see molecular changes at the certain anatomic structure and/or cells^19–21^. Circulating cell-free tumor DNA testing has been used in cases wherein the primary tumor tissue is difficult or impossible to obtain. It has also been used in follow-up tests after treatment. It can also be used in early cancer diagnosis and/or cancer screening^22–25^. Circulating tumor cell test has been used in identifying tumor cell in blood circulation, which might be used in predicting tumor metastasis and/or tumor recurrence^26,27^. Although these technologies are more often used in solid tumor testing, it might find use in the identification of molecular changes in some myeloid neoplasms, such as myeloid sarcoma, and in hematopoietic microenvironment, such as in the bone marrow.

## Examples of AML mutation tests

### APL

APL is an AML with t(15;17) translocation that leads to a chimeric gene (*PML–RARA*) on the long arm of derivative chromosome 15. APL consists of 5%–8% of all AML cases. APL is often associated with disseminated intravascular coagulation. Coagulopathy is associated with significant death rates in APL patients^1^. However, APL responds very well to tretinoin treatment and may result in a favorable prognosis, better than other AML cytogenetic subtypes^1^. Therefore, prompt and correct diagnosis is critical. Translocation can usually be identified using karyotyping. However, karyotyping takes time to generate data and results. Very often, it is not quick enough for early patient treatment decision. This is one of the reasons why the guideline allows tretinoin treatment for suspicious APL until proven otherwise. Furthermore, conventional karyotyping can only detect approximately 85%–90% of t(15:17) translocation and leave cryptic translocation undetected^28^. FISH assay can have shorter turnaround time. Different FISH assays have been developed to detect t(15;17) translocation, for example, the dual-color dual-fusion FISH assay^29^. Since FISH assay usually has shorter turn around time than karyotyping, it is very helpful in early diagnosis of APL. The dual-color dual-fusion assay uses two probes, one for PML at chromosome 15q24 and the other one for *RARA* at chromosome 17q21. Each probe overlaps the breakpoint on each chromosome respectively. Such design allows the assay detect *PML–RARA* effectively. However, in some *RARA* translocation cases, the translocation happens between the *RARA* gene and other partner genes, which poses a challenge for the dual-color dual-fusion FISH assay because the probe for PML gene would not contribute to the detection of the translocation. Another FISH assay, *RARA* dual-color break-apart FISH assay, has been designed for such a situation. The *RARA* dual-color break-apart FISH assay uses two probes for the *RARA* gene, each labeled with a different fluorophore. For example, one *RARA* probe is labeled with a red fluorophore and the other with a green fluorophore. One of the probes binds to the portion of the *RARA* gene centrometric to the breakpoint and the other to the *RARA* gene telemetric to the breakpoint. Such design allows the FISH assay to detect *RARA* translocation regardless of the partner genes. FISH assay has been widely used in APL diagnosis. Its quick turnaround time offers timely diagnosis, enabling early treatment and, consequently, resulting in a better outcome. However, FISH assay also has its own shortcomings. Although FISH assay is able to detect some cryptic translocations, some rare cryptic *RARA* translocation cannot even be detected by FISH. Therefore, PCR assays have also been designed to detect *PML–RARA* translocation. PCR assay can be used to detect cryptic *RARA* translocations^28,30–32^.

In a *PML–RARA* PCR assay, the primer design depends on where the breakpoints of each translocated gene. The breakpoints in the *RARA* gene have been seen in introns 1, 2, and 3. The majority of the breakpoints are in intron 2. Therefore, the primer design for the *RARA* gene is focused on intron 2. The breakpoints in the PML can occur at three different locations, resulting different sizes of transcripts of *PML–RARA*. The first breakpoint (BCR1) at intron 6 will result in a long transcript (also called type B). The second breakpoint (BCR2) at exon 6 will result in variable transcripts, which can be of different sizes in different patients and, therefore, are called variable form (also called type C). The third breakpoint (BCR3) at intron 3 will result in a short transcript (also called type A)^32–35^. Normally, different primers are needed to target different isoforms due to the different breakpoints on PML. Since PCR assay is usually more sensitive than karyotyping and FISH assay, it usually works better in monitoring MRD. Generally speaking, real-time quantitative reverse transcription PCR assay is used to detect fusion products at the RNA level. However, it has been reported that DNA Q-PCR can be designed to detect fusion gene in peculiar cases using patient-specific primers^36^. For PCR testing, a molecular laboratory should indicate the assay sensitivity. Most clinical laboratories have a sensitivity level of 10^−4 6^.

There are many recurrent genetic changes in AML, including translocations. The NGS assay is also an effective way of detecting translocations in AML. There are different ways to design the NGS assays. It usually uses RNA as testing material. The extracted RNA is usually transcribed into cDNA, which is used as the target for sequencing. Adapters are usually added to the cDNA, which is amplified using PCR. Molecular barcodes can be incorporated into the adapter to improve the sensitivity and specificity of the NGS assay. The targeted cDNA is captured using gene-specific probes and is sequenced later. The sequencing reads associated with translocation genes are analyzed using NGS software to detect the fusion genes. Such an assay can detect the fusion genes that are targeted during hybridization capture regardless of the partner genes. The advantage of the NGS fusion gene assay is that it can detect many translocations in one assay. It is less affected by different breakpoints. However, if the breakpoints of the fusion genes are out of the coding region, a special design targeting the non-coding region will be needed.

### AML with t(8;21) translocation

AML with t(8;21) translocation leads to a transcriptionally active chimeric gene on the 8q-derivative chromosome (*RUNX1–RUNX1T1*)^16^. Such a translocation can usually be detected using karyotyping. However, karyotyping may have false-negative result if the cells with the translocation fail to proliferate during the assay or the translocation is cryptic. FISH assay on interphase cells has been developed to solve this problem^16^. The dual-color FISH test is able to interrogate interphase cells and detect some cryptic translocation. Therefore, it is more sensitive than karyotyping. The tests for t(8;21) translocation help to identify this AML category, which normally has a high rate of complete remission and favorable outcome. Occasionally, the blasts in this type of AML may be less than 20%. However, when t(8;21) is present, less than 20% of blasts should not invalidate the diagnosis of AML.

### AML with CEBPA mutation

One of the subtypes of AML is AML with biallelic *CEBPA* mutation. It has been reported in 4%–9% of children and young adults with AML^1^. *CEBPA* is a one-exon gene. *CEBPA* mutations can happen at almost any part of the gene. Therefore, a sequencing assay is more suitable to detect mutations in *CEBPA* gene. Sanger sequencing is a gold standard sequencing method. Sanger sequencing can effectively detect mutations in *CEBPA* gene. As mentioned before, the lower detection limit is about 15%–20% VAF. Although *CEBPA* mutation can be germline mutations, many *CEBPA* mutations are somatic. Therefore, for a sample to have a mutation with 15%–20% VAF, there should be at least 30%–40% of leukemic cells in the sample, assuming all the leukemic cells have somatic mutation. In other words, if a sample has less than 30%–40% of leukemic cells and is tested negative, a false-negative result cannot be ruled out.

The subtype of AML with *CEBPA* is defined by having bi-allelic *CEBPA* mutations. In reality, when two mutations are found in a sample, the data may or may not be able to tell if the mutations are bi-allelic. If the data show that two mutations are on the same sequencing strand, we know that the two mutations are not bi-allelic. Otherwise, we are unsure. In general, when two *CEBPA* mutations are detected, it is assumed to be bi-allelic unless proven otherwise.

NGS can also be used to detect *CEBPA* mutation. However, *CEBPA* gene sequencing can be challenging due to high GC content. This is especially challenging for an amplicon NGS assay. Sometimes, an amplicon NGS assay can only detect about 50% of the *CEBPA* mutations. For example, in one amplicon assay, effort has been made to get good coverage on the *CEBPA* gene. Six pairs of primers have been designed to cover both the + and – strands of *CEBPA* to cover the entire *CEBPA*. However, actual data show that 56% of the gene is covered with less than 200 reads. Therefore, if an amplicon NGS assay generates a negative test result, one needs to ensure that the coverage is appropriate. Otherwise, a false-negative result cannot be ruled out. Hybridization capture NGS assay works better than amplicon assay in sequencing *CEBPA* gene for several reasons. First of all, the DNA fragment process in the hybridization assay is not affected by the GC content in targeted DNA. The second, since the fragmentation is almost random, the sequencing reads overlap with each other better and are not affected by the GC content in the targeted DNA, which results in a more even coverage along the full gene sequence. Therefore, if an NGS assay is to be used for *CEBPA* mutation detection, it will be better to use hybridization capture assay. An appropriate use of molecular tests will help to identify this group of AML, which has a relatively low risk.

### AML with FLT3 mutation

*FLT3* mutations have been seen in approximately 30% of AML^37–39^. In general, the *FLT3* mutation is considered as one of the driver mutations, but it is not used to define a subtype of AML. *FLT3* mutations are usually associated with unfavorable prognosis^6,7,40^. *FLT3* inhibitors have been developed for the treatment of the AML^41–43^. The most common *FLT3* mutations are either *FLT3* internal tandem duplicate (*FLT3*–*ITD*) repeats or tyrosine kinase domain point mutations (*FLT3*–*TKD*). The biological impacts of these two types of mutations could be different. More data have linked *FLT3–ITD* to unfavorable prognosis. The ratio of *FLT3–ITD* mutant and *FLT3* wild type has been adopted in AML diagnosis and management guideline^7^.

Different methods have been used to detect *FLT3* mutations^44–48^. One method uses PCR to amplify *FLT3* and uses capillary gel fragment analysis to measure the length of the PCR products^47^. The PCR products of *FLT3*–*ITD* are longer than that of the wild type. The peak height is somewhat proportional to the amount of PCR product and, therefore, reflects the amount of mutant or wild type. The peak height is used to calculate the ratio of the *FLT3–ITD* mutant to the wild type, which is used in the treatment guideline. With some modifications, the *FLT3–TKD* can be detected with this method. The most common *FLT3–TKD* mutation is at *FLT3 D835*. The *D835* wild-type gene happens to be within an *Eco*RV restriction endonuclease cutting site. Therefore, the wild-type *FLT3* is susceptible to *Eco*RV digestion. Meanwhile, a mutation at *FLT3 D835* will eliminate this *Eco*RV restriction site and *FLT3* is no longer susceptible to *Eco*RV digestion. After *Eco*RV digestion, the *FLT3–TKD* and wild-type *FLT3* will generate PCR products of different sizes, which can be identified using fragment analysis. With such an assay design, both *FLT3–ITD* and *FLT–TKD* can be detected in the same multiplex PCR and fragment analysis assay.

*FLT3–TKD* can also be easily detected using NGS assay. *FLT3–ITD* mutations, however, pose a challenge to a regular amplicon NGS assay due to variable sizes of the ITDs, some of which could be more than 150 bp long. Hybridization NGS could be a better method than the regular amplicon assay^49^. With appropriate assay design and pipeline design, such NGS assay should be able to detect most of *FLT3–ITD*. The VAF obtained in NGS assay can be used to calculate the ratio of *FLT3–ITD vs* wild-type *FLT3*, using the following formula: ratio = VAF/1 − VAF. The ratio obtained in this way should be very close to the ratio obtained from PCR fragment analysis assay, which is adopted by the risk stratification guideline. However, there is a caveat. In NGS assay, different filters have been set up. The filters are intended to filter non-specific false signals, but that is not always the case. The filters sometimes may filter specific real signals. It is a tradeoff between sensitivity and specificity. In case of deletion and insertion, NGS assay tend to underestimate VAF due to filters. It might be better to use BAM file VAF to calculate *FLT3–ITD vs FLT3* wild-type ratio. VAF from BAM file has gone through fewer filters and might be more closely approximating the ratio obtained from traditional PCR assay. Since the difference between more or less filters is a tradeoff of sensitivity and specificity, a molecular laboratory should make a balance decision based on validation data.

It has been suggested that *FLT3–ITD* be used as a marker of MRD for *FLT3–ITD*–positive AML^50–52^. Although there are pros and cons about the use of *FLT3–ITD* as a marker for MRD, efforts have been made to develop tests for it. NGS assay has been explored for this application. By increasing the depth, using unique molecular barcode, and employing special design of bioinformatics software, different NGS assays have been published with different sensitivities in measuring *FLT3–ITD* for AML MRD^49,53–55^. An appropriate use of molecular tests will help to identify this high-risk factor in AML patients.

### Tests for MRDs

It has been realized that the ability to identify AML MRD is important for risk stratification and prognosis^56^. Currently, MRD means the presence of leukemia cells down to levels of 1:10^4^ to 1:10^6^ white blood cells (WBCs)^56^. Evidently, there is a 100 times difference between 1:10^4^ and 1:10^6^. The definition of MRD will be refined as more data become available. Both flow cytometry and molecular tests can be used to measure MRD. Here, we focus on molecular tests.

Many different gene mutations have been identified in AML. Some of these mutations may be used for MRD tests while the other may not. For example, *NPM1* mutations and the fusion genes *RUNX1–RUNX1T1*, *CBFB–MYH11*, and *PML–RARA* have prognostic value and therefore could be used as markers for MRD tests^56^. Meanwhile, *DNMT3A*, *ASXL1*, and *TET2* gene mutations are not correlated well with prognosis and tend to occur in healthy individuals as they age. Therefore, these mutations may not be good molecular markers for AML MRD tests^57–60^. Because of AML clonal evolution, there are frequent losses and gains of gene mutations during relapses. Therefore, some gene mutations may not be good candidates as sole molecular marker for MRD tests, e.g., *FLT3–ITD*, *FLT3–TKD*, *NRAS*, *KRAS*, *IDH1*, *IDH2*, *MLL–PTD*. However, as a group, these mutations may be used as molecular markers for MRD test, especially when used in combination with a second MRD marker^56^. Clinical situations should also be considered when selecting molecular markers for MRD tests. In general, germline mutations in *RUNX1*, *GATA2*, *CEBPA*, *DDX41*, and *ANKRD26* may not be good candidates for MRD tests. However, in allo-hematopoietic stem-cell transplantation patients, germline gene mutations may be useful molecular markers. Another example is WT1 expression. The ELN MRD working group indicates that WT1 expression should not be used as an MRD marker since it has low sensitivity and specificity, unless no other MRD markers, including flow cytometric ones, are available in the patient^56^.

Depending on the molecular markers selected, different molecular assays can be used. When a single-gene mutation is selected as MRD marker, traditional real-time quantitative (RQ) PCR is a useful technology. For example, *NPM1* mutation can be tested using RQ-PCR^61^. Digital PCR assay is a relatively new technology that can be used for *NPM1* quantitative test. A massively multiplex ddPCR assay has been published, which can also detect different types of *NPM1* mutations^62^. When multiple gene mutations are used as MRD markers, NGS technology has advantages^63,64^. The application of UMI barcode has reduced NGS error and noise background, which has paved the way for NGS application in MRD testing^9^.

### Tests for clonal evolution

Clonal evolution is a well-known fact. NGS assays are good methods in obtaining information about the clonal evolution in any given patient. Single-cell NGS assay is even more informative in obtaining information about the clonal evolution^17,18^. The new technology has paved the way for a better personalized precision medicine in the future.

In the future, AML molecular test will continue to capitalize on NGS technology. Current myeloid NGS panel will be improved in several aspects. One aspect is to cover more relevant genes discovered by recent scientific research. As NGS technology advances and the cost of NGS decreases, it may become feasible to use whole-exome sequencing in routine AML clinical testing. The second aspect is to improve the sequencing coverage for those hard-to-sequence genes. For example, some genes have a high CG content, which posts challenge to NGS sequencing. Other genes have large insertions and/or deletions, which are also challenging for NGS sequencing. Some genes not only have large insertions, but also have variable insertion sizes, which makes these genes even more challenging for NGS sequencing. The improvement on wet laboratory, such as combining amplicon and hybridization technology, and the improvement on bioinformatics are being made to overcome such challenges. The third aspect is to improve NGS non-coding region sequencing. The importance of this has been realized in myeloid neoplasms, especially for those genes associated with myeloid neoplasms with germline predisposition. The fourth aspect is to improve NGS for translocation detection. In short, an NGS assay, which covers all gene mutations, including point mutations, insertions, deletions, relevant non-coding region mutations, and translocations, will become feasible in routine clinical testing. Apart from the above, epigenetic change is gaining more and more attention.

Traditional single-gene molecular assays will continue to play an important role in AML testing, complementing NGS assay. Generally speaking, single-gene testing offers quick turnaround time and lower cost. Traditional single-gene tests can be used in follow-up patients to test the gene mutations that are known in a given patient. For example, real-time PCR assay can be used in MRD follow-up test for certain known mutations in certain patients.
